# The impact on the scientific community of the 2018 addendum to the CHCC

**DOI:** 10.3389/fmed.2022.1081063

**Published:** 2022-12-01

**Authors:** Marzia Caproni, Valentina Ruffo di Calabria, Elena Biancamaria Mariotti, Alice Verdelli, Cristina Aimo, Alberto Corrà, Lavinia Quintarelli, Walter Volpi, Erkan Alpsoy, Cord Sunderkötter

**Affiliations:** ^1^Immunopathology and Rare Skin Disease Unit, P.O. Piero Palagi, Azienda USL Toscana Centro, ERN-SKIN Member, University of Florence, Florence, Italy; ^2^Section of Dermatology and Venereology, Department of Health Sciences, University of Florence, Florence, Italy; ^3^Section of Dermatology and Venereology, Azienda USL Toscana Centro, Florence, Italy; ^4^Section of Dermatology and Venereology, Akdeniz University School of Medicine, Antalya, Turkey; ^5^Department of Dermatology and Venereology, University Hospital Halle (Saale), Martin-Luther-University Halle-Wittenberg, Wittemberg, Germany

**Keywords:** vasculitides, nomenclature, Chapel Hill Consensus Conference, addendum to Chapel Hill Consensus, provisional definitions of vasculitides

## Introduction

The term vasculitis encompasses a wide and heterogeneous group of disorders with shared histopathological findings, namely inflammation and necrosis of the blood vessel wall with variable hemorrhagic and ischemic features. Vasculitis may range in severity from a self-limited disorder in one single organ to a life-threatening disease as vessels of any size can be affected (1).

The 1994 Chapel Hill Consensus Conference Nomenclature of Vasculitides (CHCC1994) managed to provide a consensus on definitions of such conditions instead of a classification system or shared diagnostic criteras. At first, vasculitides were distinguished according to the size of the affected vessels and the involved immunopathogenic process. Because of the scientific advances in the understanding of the underlying mechanisms, the classification was further expanded and updated with the 2012 revised International CHCC (CHCC2012), which resulted in the introduction of new categories namely Variable vessel vasculitis, Vasculitis associated with systemic disease and Vasculitis associated with probable etiology (2).

However, even though vasculitides frequently involves the skin, it was only in 2018 that a standardized specific nomenclature was proposed on the basis of the CHCC nomenclatures to highlight the special features of cutaneous vasculitides (CV). These acquirements were included in the Dermatologic Addendum to CHCC2012 (D-CHCC). Accordingly, CV were divided into three groups: (1) CV as part of a systemic vasculitis; (2) skin-limited or skin-dominant vasculitis as a variant of a systemic vasculitis which is restricted to the skin without clinically visible or manifested systemic vasculitis and (3) single organ vasculitis of the skin (SOV). The SOVs group has no equivalent in other organs and they differ from the skin-dominant forms since they do not fulfill sufficient clinical, laboratory, and/or pathologic features of a known systemic vasculitis. They encompass Nodular vasculitis (erythema induratum of Bazin), Erythema elevatum et diutinum, Recurrent macular vasculitis in hypergammaglobulinemia (hypergammaglobulinemic purpura of Waldenstrom) and Normocomplementemic urticarial vasculitis (3). However, other forms, e.g., IgM/IgG immune complex vasculitis, may be also included in this group in the future if supported by research. A good example of systemic vs. skin-limited vasculitis would be IgA vasculitis (IgAV), since many patients with vasculitis which present at dermatology offices have skin-limited IgA: the latter is confirmed by leukocytoclastic vasculitis on histopathology and perivascular IgA deposition on immunofluorescence. While these patients do not show pathological urine, i.e., no erythrocytes urine and no abdominal pain as well as absence of signs of nephritis (non-pathological urine analysis, no dysmorphic erythrocytes, no rise in blood pressure), of gastrointestinal vasculitis (no postprandial abdominal pain, negative hemoccult) and of arthritis, though one cannot exclude that they would show microscopic alterations such as IgA deposition in kidneys since one would not subject these patients to renal biopsies. Systemic but also skin limited IgAV present with perivascular deposition of hypogalactosidated IgA (GdIgA), so this modified IgA1 is not the reason for the difference (4), but patients with systemic IgAV appear to have higher serum levels of GdIgA during active disease (5, 6). None of these Consensus conferences was ever meant to provide diagnostic criteria, but rather to standardize an expanding terminology of different nosologic entities. In more recent years the Diagnostic and Classification Criteria in Vasculitis (DCVAS) represented an interdisciplinary attempt to implement the classification criteria of systemic vasculitides by recruiting 6,991 participants from 136 sites in 32 countries starting from January 2011 to December 2017 (7). The extensive data set collected internationally thanks to the DCVAS study has been subsequently analyzed and resulted in the 2022 American College of Rheumatology/European League Against Rheumatism (ACR/EULAR) classification for Microscopic polyangiitis (MPA), Granulomatosis with Polyangiitis (GPA) and Eosinophilic Granulomatosis with Polyangiitis (EGPA) (8, 9) These new formal criteria are based on weighted items, including also ANCA testing and modern imaging techniques. Because of their excellent sensitivity and high specificity, they represent a useful tool for the clinician, in the setting of clinical research, in differentiating cases of MPA/GPA/EGPA from similar types of vasculitides, when a diagnosis of small- or medium-vessel vasculitis has already been made and other conditions that potentially mimic vasculitides have already been excluded. It is important to note once more that even though these criteria are not meant for diagnostic purpose in the everyday clinical practice, they have been validated to replace the European Medicines Agency (EMA) algorithm published in 2007, previously used to harmonize and rationalize the use of the ACR and CHCC classification systems for epidemiologic purposes (10).

Finally, Diagnostic and Classification criteria in VASculitis (DCVAS) also developed diagnostic criteria for ANCA Associated Vasculitis (AAV) in a large study recruiting patients into an international cohort from 2010 until December 2017 with AAV and comparator diseases (11–13).

## The impact of dermatological addendum in scientific community

**Figure 1:**
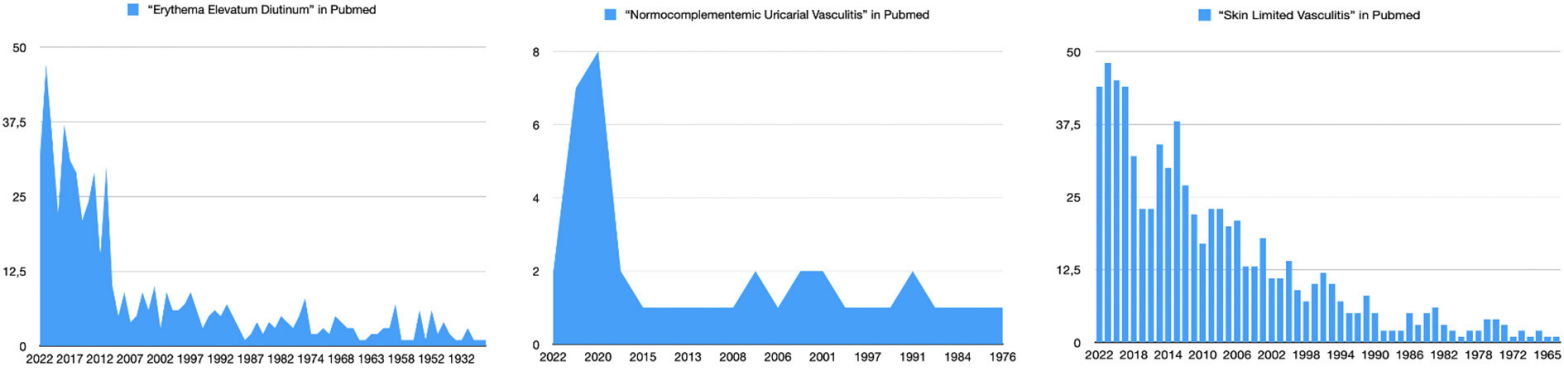
Count of the articles that used the terms “Normocomplementemic Urticarial Vasculitis”, “Erythema Elevatum Diutinum” and “Skin Limited Vasculitis” over the years in Pubmed.

We performed a review of the literature to evaluate the actual impact that the D-CHCC has had during the last 4 years in the scientific community. From 2018 until September 2018, the Addendum has been cited in 115 publications on Pubmed and the number of citations per year showed an increasing pattern with a maximum peak reached in 2021 (*n* = 36) (Figure 1). Of these publications, 107 (1, 2, 4, 6, 14–115) were written in English and published by Journals specialized in Dermatology (*n* = 41), Rheumatology (*n* = 20) and Immunology (*n* = 13) and their Countries of publication were mainly represented by the United States of America (*n* = 37), Germany (*n* = 21), United Kingdom (*n* = 16).

Limiting the research to the new described CV, we only found one study in the English literature that was aimed to further investigate the clinical and immunopathologic features of cutaneous IgM/IgG immune complex vasculitis (8) Its main goal was to elucidate the clinical differences between IgM/IgG vasculitis and the more common skin-limited IgA vasculitis (sI-IgAV), thus proposing practical advice for the everyday medical practice. Hemorrhagic blisters and targetoid lesions seemed to be more frequent in the sI-IgAV group, suggesting that these two features could represent a valuable clinical tool that may help differentiating the two forms when immunological tests are unavailable or unaffordable. A group of researchers tried to link special clinical characteristics to the “Recurrent macular vasculitis in hypergammaglobulinemia” category in 2019, while this term was hardly used before 2018 and the clinical symptoms or very similar diseases were referred to with many different names (golfer's vasculitis, Waldenströn purpura, exercise-induced vasculitis, Saturday night vasculitis). Concerning “Normocomplementemic urticarial vasculitis,” a slight increase in its usage was appreciable after the introduction of the D-CHCC. In fact, before 2018, eight articles only reported this term over 27 years (1991–2018), while after 2018, nine cases could be counted until now (Figure 1). The “Erythema elevatum et diutinum” category, although largely adopted throughout the years, reached its peak of use the biennium 2019–2020 (Figure 1).

Extending our search to other forms of vasculitis, such as IgA vasculitis, we found many discrepancies. In fact, the term “Henoch-Schonlein purpura” has been used 962 times, mainly by Pediatrics journals, instead or together with the updated and more accurate terminology of “IgA vasculitis.” These data show that there is still confusion in the use of terminology, often based on old classifications and highlight the need for a synergistic work to reach a real widespread consensus on the nomenclature of vasculitis. Moreover, only a few articles (48 in 2021) use the term “skin limited vasculitis” to distinguish these forms from the ones with systemic involvement, although its number has risen since publication of D-CHCC (Figure 1). Thus, although this phenomenon has been known for a long time by dermatologists, it is still not reflected by a punctual use of the specific terms.

## Discussion

The D-CHCC has been adopted internationally by several experts of the field. According to our data, many authors have cited the D-CHCC in their work in the last years as it has been quoted almost 200 times so far, thus recognizing its clarifying role in the nomenclature of CV. Many are becoming more familiar with the new terminology, mainly dermatologists and rheumatologist and especially experts in the field of vasculitis or dermatopathology have explicitly welcome its appearance and supported its use. However, it is not yet mentioned in all articles on CV and several recent publications on vasculitis do not use the terminology as it was consented on. In this regard, it is not of secondary importance the still widespread use of eponymous terminology. This issue is linked to that of the provisional definitions or unsolved problems in the actual nomenclature. For example, while the term IgG/IgM vasculitis will likely continue to be used, further data must be provided for the existence of an isolated form of IgG/IgM vasculitis (no IgA involvement; no other, IgG-IC generating autoimmune disorders associated) (56). The establishment of a standardized and universally accepted nomenclature will also provide a fundamental base for multicentric studies, which would allow us to collect and compare a more significant set of data that is nowadays lacking for a deeper understanding of CV. As modifications to the actual nomenclature are possible, dermatologists and rheumatologists, among others, are invited to contribute with suggestions for improvement. In fact, the D-CHCC represent a basis for interdisciplinary discussion on CV offering written statements which can be either falsified or verified by clinical observation on patients. One of the aims of the D_CHCC group was the chance to test the practicability of the D-CHCC definitions in the clinical setting. In particular it would be useful to know, if each dermatosis with histopathologically proven vasculitis, can be assigned to the various defined forms of vasculitides. Another aim of the D-CHCC was to encourage the acquisition of new data to help clarify e.g., the existence of lymphocytic or eosinophilic vasculitis according to the consented, but provisional definitions.

To conclude, CV encompass a wide and heterogeneous group of rare conditions, probably often underdiagnosed and under investigated by clinicians with no specialized dermatologic competences, especially in the cases where clinical manifestations are minor and self-limiting. Even though some clinical entities are now known to have specific clinical features, much more needs to be done to further implement our knowledge in the underlying pathogenetic mechanisms, which remain partially unknown and is of fundamental importance for a better diagnostic and therapeutic management of patients suffering from CV.

## Author contributions

MC, EA, and CS conceptualized the work. VC and EM wrote the text. AV and CA collected the data. AC, LQ, and WV revised the drafts. All authors contributed to the article and approved the submitted version.

## Conflict of interest

The authors declare that the research was conducted in the absence of any commercial or financial relationships that could be construed as a potential conflict of interest.

## Publisher's note

All claims expressed in this article are solely those of the authors and do not necessarily represent those of their affiliated organizations, or those of the publisher, the editors and the reviewers. Any product that may be evaluated in this article, or claim that may be made by its manufacturer, is not guaranteed or endorsed by the publisher.
